# Performance of broad-spectrum targeted next-generation sequencing in lower respiratory tract infections in ICU patients: a prospective observational study

**DOI:** 10.1186/s13054-025-05470-z

**Published:** 2025-06-04

**Authors:** Chuanxi Chen, Ruizhi Wang, Bilin Wei, Yili Chen, Dejian Gu, Jiangze Xu, Huifang Zheng, Zimeng Xu, Linfang Ding, Xiaonan Chen, Lihua Xiao, Liping Bai, Zimeng Liu, Yongjun Liu, Minying Chen, Peisong Chen, Xiangdong Guan, Jianfeng Wu

**Affiliations:** 1 Department of Critical Care Medicine, The First Affiliated Hospital, Sun Yat-Sen University, No.58 Zhongshan Er Road, Guangzhou, Guangdong China; 2 Guangdong Clinical Research Center for Critical Care Medicine, Guangzhou, Guangdong China; 3 Department of Laboratory Medicine, The First Affiliated Hospital, Sun Yat-Sen University, No.58 Zhongshan Er Road, Guangzhou, Guangdong China; 4 Advanced Medical Technology Center, The First Affiliated Hospital, Zhongshan School of Medicine, Sun Yat-Sen University, Guangzhou, Guangdong China; 5 Geneplus-Beijing Co., Ltd., Beijing, China; 6 Department of Laboratory Medicine, Yingjiang County People’s Hospital, Dehong, Yunnan China

**Keywords:** Lower respiratory tract infections, tNGS, mNGS, Capture probe enrichment, Pathogen

## Abstract

**Purpose:**

Targeted next-generation sequencing (tNGS) has emerged as an advanced diagnostic technique. While tNGS is increasingly recognized as a valuable tool for detecting infections, its most relevant clinical indications remain underdefined. This study aimed to evaluate the clinical utility of tNGS for lower respiratory tract infections (LRTIs).

**Methods:**

We conducted a prospective, observational study to evaluate the clinical diagnostic value of broad-spectrum targeted Next-Generation Sequencing (bstNGS) covering 1872 microorganisms in critically ill patients with LRTIs. We compared the microbial detection performance of bstNGS, mNGS, and traditional culture methods in bronchoalveolar lavage fluid (BALF). Additionally, we used the odds ratio (OR) from multiple logistic regression to assess the impact of relevant clinical variables on the detection of pathogens by bstNGS. We also examined the correlation between bstNGS pathogen detection results and clinical outcomes.

**Results:**

Between August 23, 2023, and April 24, 2024, samples from 150 patients were analyzed. bstNGS detected 96.33% and 91.15% of the microorganisms discovered by mNGS and culture respectively, and was capable of identifying microorganisms with even lower loads. According to the diagnostic criteria, bstNGS, mNGS, and culture methods detected pathogens in 87.33%, 82.00%, and 46.00% of the samples respectively. Moreover, the NGS methods demonstrated a stronger pathogen detection ability compared to culture (*p* < 0.05). Further comparing the diagnostic performance of the three methods, bstNGS exhibited higher diagnostic accuracy than both mNGS (90.67% vs 86.00%, *p* < 0.05) and culture (90.67% vs 49.33%, *p* < 0.0001). Multivariate analysis revealed that immunocompromise was associated with a lower efficiency of pathogen detection by bstNGS (*p* = 0.04), while other included clinical features had no significant correlation with bstNGS detection. Additionally, compared with patients in whom no pathogen was detected, patients in whom a pathogen was detected by bstNGS were associated with better outcomes of antibiotic treatment (89.68% vs. 62.50%; OR 7.53, 95% CI 1.41–45.30; *p* = 0.02).

**Conclusion:**

This study shows the effectiveness of bstNGS in detecting pathogens of LRTIs, as well as its value as a potential auxiliary diagnostic method in the ICU.

**Supplementary Information:**

The online version contains supplementary material available at 10.1186/s13054-025-05470-z.

## Introduction

Lower respiratory tract infections (LRTIs) represent a major global health concern, particularly among patients in intensive care units (ICUs), who often present with severe conditions and compromised immune systems. As a result, the mortality rate among these patients can reach as high as 50% [1–3]. Rapid and accurate infection control is essential for improving patient outcomes in such cases [4]. The etiology of LRTIs is complex, involving a range of pathogens, including bacteria, viruses, and fungi, which complicates clinical differentiation [2, 3]. Furthermore, conventional diagnostic methods, such as microbial cultures and galactomannan (GM) testing, are not only time-consuming but also have limited sensitivity [4, 5]. Consequently, there is a growing need for more effective pathogen detection methods.

Metagenomic next-generation sequencing (mNGS) is a technology capable of identifying virtually any microorganism in clinical specimens. This method, which does not require prior specification of pathogens, has provided unexpected insights into clinical diagnostics [6–8]. However, mNGS is expensive, and its detection accuracy can be hindered by the presence of host nucleic acids, resulting in unstable detection of certain microorganisms and antimicrobial resistance (AMR) genes [9]. To address these limitations, two promising strategies for improving mNGS have emerged: host nucleic acid elimination and targeted enrichment of microbial nucleic acids [10, 11].

Targeted next-generation sequencing (tNGS), a variant of mNGS, has been developed to address these challenges [12]. Several studies have highlighted the advantages of target enrichment for pathogen identification in various infectious diseases, including severe pneumonia [13–15]. While the diagnostic value of tNGS has been well described in retrospective comparisons with conventional diagnostic methods, few prospective studies have assessed its performance. As such, the role of tNGS within the broader diagnostic toolkit remains to be fully clarified. tNGS generally covers dozens or hundreds of microorganisms, we introduced a broad-spectrum tNGS (bstNGS) upgraded to include 1872 microorganisms, expecting to reduce the diagnostic insufficiency caused by the scope of targets.

Therefore, this prospective study aims to assess the performance of broad-spectrum targeted next-generation sequencing (bstNGS) in ICU patients with LRTIs. Additionally, we examined the factors influencing pathogen detection by bstNGS and its association with patient outcomes.

## Methods

### Sample enrollment and standard-of-care microbiology testing

**Fig. 1:**
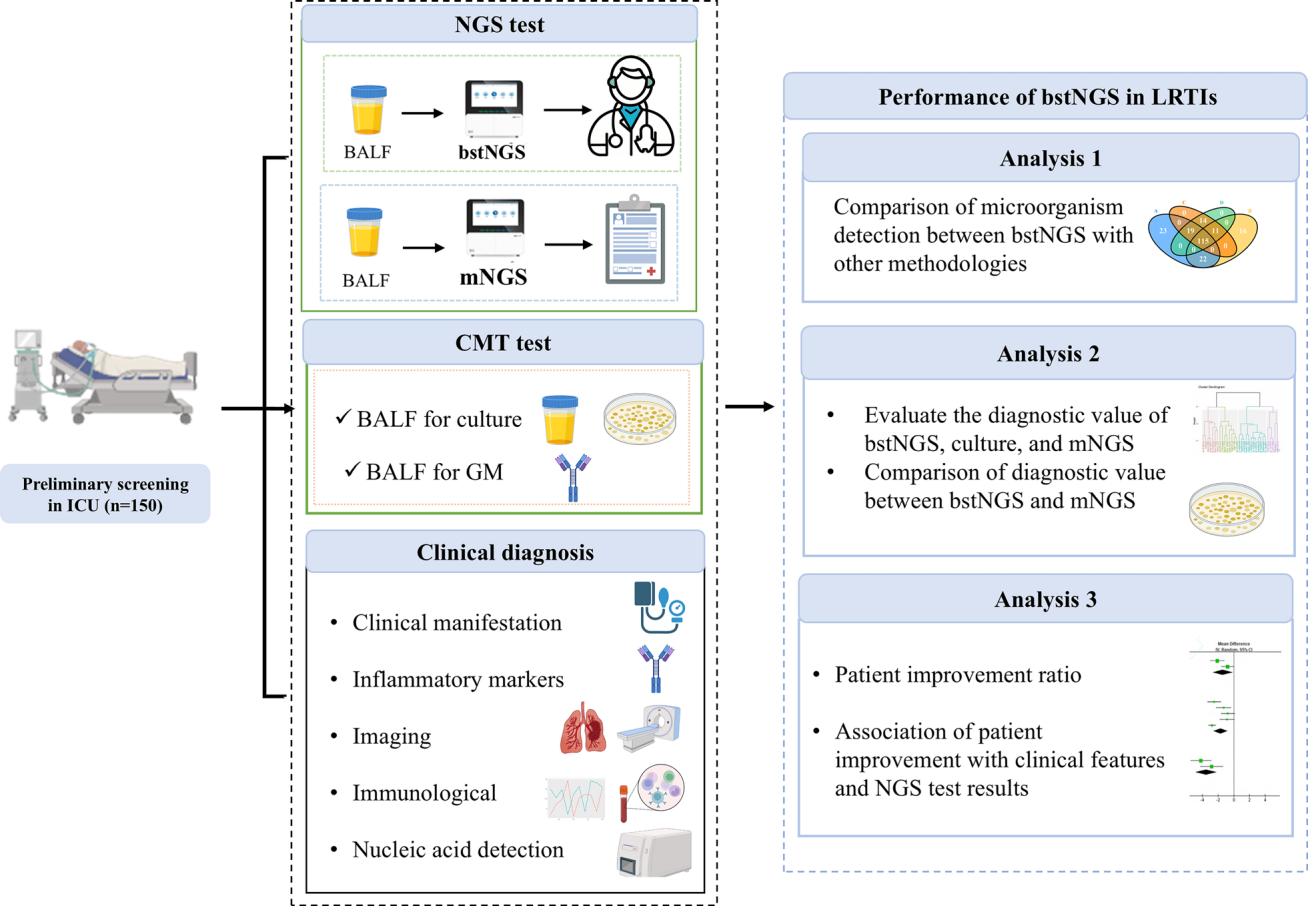
Flowchart of patients enrolled and the bstNGS workflow. Patients with Lower respiratory tract infections (LRTIs) symptoms treated in the intensive care units (ICU) were included in this study. Pathogenic microorganisms were determined using various methods

From August 23, 2023, to April 24, 2024, we prospectively enrolled patients with suspected lower respiratory tract infections (LRTI) who were admitted to the ICU at The First Affiliated Hospital of Sun Yat-sen University (Fig. 1). The study protocol was approved by the local ethics committee, and informed consent was obtained from the patients or their legal representative. This research adhered to the principles outlined in the Declaration of Helsinki and was registered with the Chinese Clinical Trials Registry (ChiCTR2300074119) on July 31, 2023. Patients were eligible for enrollment if they: (1) were aged ≥ 18 years; (2) were admitted to the ICU due to suspected LRTI; and (3) had bronchoalveolar lavage fluid (BALF) samples available for testing. Suspected LRTI was defined by the presence of new or progressive pulmonary infiltrates on chest radiographs and at least one of the following criteria: (1) body temperature > 38 °C or < 36 °C; (2) leukocytosis > 12,000/mm^3^ or leukopenia < 4000/mm^3^; (3) exacerbation of cough, sputum, or respiratory symptoms, with or without chest pain, dyspnea, or hemoptysis. Patients were excluded if they had been diagnosed with non-infectious pneumonia prior to enrollment or if their life expectancy was less than 24 h. The identification of immunocompromised patients was based on previous studies [16, 17].

### Conventional microbiological tests

BALF samples were collected from all patients within 48 h of ICU admission for conventional microbiological testing, including mNGS and bstNGS. The conventional microbiological tests included bacterial, mycobacterial, and fungal cultures, as well as detection of galactomannan (GM) (Platelia™, Bio-Rad, France) in BALF. Following the introduction of mNGS into the study, viral PCR tests were no longer performed.

### Broad-spectrum tNGS assay and mNGS workflow

The details of the bstNGS procedure are provided in the Supplementary Methods. As described in previous studies [17, 18], the bstNGS assay involved DNA and RNA extraction, library construction, and enrichment using Geneplus probes, which cover 1872 species, including 1124 bacterial species, 218 fungal species, 474 viral species, and 56 parasitic species (Supplementary Table 1). The enrichment process lasted for 4 h. Sequencing was conducted on the Gene^+^ Seq-100 platform (GenePlus, Suzhou, China), generating single-end 100 bp reads, with a total of 5 million reads. After sequencing, ensure that the final sequencing output is no less than 5 million reads, with Q20 and Q30 values not lower than 95% and 88%, respectively. Clean reads were obtained by removing sequencing adapters, low-quality reads, or reads below 35 bp using fastp (version 0.23.1) [19]. The filtered reads were compared with the self-built pathogenic microorganism database using Burrows-Wheeler Aligner software (BWA, 0.7.12-r1039) [20], and the retained results were annotated. The reads per million (RPM) of pathogenic microorganisms were calculated. The threshold was set at RPM ≥ 6 for common pathogens (excluding mycobacteria) and ≥ 0.5 for fungi and mycobacteria. A manual review is conducted. Oral commensals were not reported regardless of their relative abundance, unless otherwise proven or deemed significant by the attending physician. Microorganisms with abnormal genomic coverage will be filtered out. In parallel with bstNGS, an aliquot of each sample was also processed for mNGS analysis, involving both DNA and RNA, as previously described [21, 22].

### Clinical diagnosis as the reference standard

Two clinically experienced intensivists (CC and JW), each with over ten years of experience in the ICU, independently reviewed the medical records, microbiological test results (including mNGS and bstNGS), and clinical data for all patients during their ICU stay. Adopt the clinical diagnostic criteria of previous studies [23–25], initially, they determined whether the patient had an infectious disease. Subsequently, they identified the pathogens from microorganisms based on clinical manifestations, laboratory tests, imaging findings, microbiological examinations (including cultures, mNGS, and bstNGS), and treatment responses. Microorganisms were classified as causative pathogens, possibly causative pathogens, or microorganisms that are unlikely to cause symptoms, and the first two were identified as pathogens related to infection (Supplementary Fig. 1).

### Outcomes

The clinical outcomes of antibiotic therapy are classified according to the ENIRRIs [26] study, with detailed criteria as follows (Supplementary Fig. 1):

- Benefit: Improvement or stabilization of infection-related signs and symptoms (e.g., white blood cell count, chest radiograph, and fever), as assessed using the Clinical Pulmonary Infection Score (CPIS). Improvement or stabilization was observed by the seventh day, particularly if chest X-ray findings showed improvement or remained stable at an acceptable level.
- Failure: No overall improvement in infection-related signs and symptoms, or deterioration in pulmonary radiography (Supplementary Fig. 1).

### Statistical analysis

Continuous variables are presented as medians with interquartile ranges (IQR), while categorical variables are expressed as frequencies and percentages. Inter-group comparisons of continuous variables were made using the unpaired t-test or the Mann–Whitney U test. Comparisons between groups of categorical variables were performed using the chi-square test. To assess diagnostic performance, sample consistency was evaluated according to previous studies (Supplementary Fig. 2) [23]. The McNemar test was used to compare the diagnostic performance of bstNGS and mNGS. Multivariate logistic regression was employed to compute the odds ratio (OR), with p-values < 0.05 considered statistically significant.

Statistical analyses were conducted using SPSS version 26.0 (SPSS Inc., Chicago, Illinois, USA). Figures were generated using GraphPad Prism (version 9.2.0) and R 4.3.1 software (http://www.r-project.org, The R Foundation). Errors are reported as 95% confidence intervals or standard deviations where indicated. A p-value < 0.05 was considered statistically significant. Thresholds for significance in correlation coefficients were adjusted for multiple comparisons using Bonferroni’s correction [27].

## Results

### General information of the patients

From August 23, 2023, to April 24, 2024, a total of 150 patients met the inclusion criteria and had sufficient samples for analysis. All patient samples underwent standard culturing, additional routine microbiological assessments, and mNGS testing. The remaining samples were analyzed using broad-spectrum targeted NGS (bstNGS) for concurrent detection. Demographic details and baseline medical conditions of the patients are summarized in Table 1. The median age of the cohort was 64 years, and 30.67% (46/150) of the patients were immunocompromised. During their ICU stay, 96.00% (144/150) of the patients received mechanical ventilation. Ultimately, 18.67% (28/150) of the patients succumbed to their conditions during their ICU stay.

**Table 1:** Clinical characteristics of patients

Clinical characteristic (n = 150)	Number (%)
Age, median (IQR)	64 (53,72)
Gender	
Male	108 (72.00%)
Female	42 (28.00%)
Laboratory findings	
WBC (10^9^/L), median (IQR)	11.82 (8.57, 16.05)
CRP (nmol/L), median (IQR)	97.08 (51.31, 152.13)
PCT (ng/mL), median (IQR)	1.32 (0.50,4.58)
Underlying diseases	
Active solid neoplasia	37 (24.67%)
Cardiovascular disease	10 (6.67%)
Chronic liver disease	6 (4.00%)
Chronic lung disease	4 (2.67%)
Gastrointestinal diseases	11 (7.33%)
Hematologic malignancies	4 (2.67%)
Neurological disease	23 (15.33%)
Traumatic disease	16 (10.67%)
Immunocompromised	
Active solid neoplasia	37 (24.67%)
Hematologic malignancies	4 (2.67%)
Solid-organ transplantation	5 (3.33%)
Disease severity	
APACHE II score, median (IQR)	21 (15, 25)
SOFA score, median (IQR)	8 (5, 11)
Septic shock	6 (4.00%)
ARDS	79 (52.67%)
Mechanical ventilation, n (%)	144 (96.00%)
CPIS, median (IQR)	6 (4, 10)
Outcome	
ICU death	28 (18.67%)
Days of ICU stay, median (IQR)	11 (6, 20)

*APACHE* Acute Physiology and Chronic Health Evaluation, *ICU* intensive care unit, *IQR* interquartile range, *SOFA* Sequential Organ Failure Assessment, *WBC* White blood cell, *PCT* procalcitonin, *CRP* C-reactive protein

### Detection capabilities of bstNGS for microorganisms

**Fig. 2:**
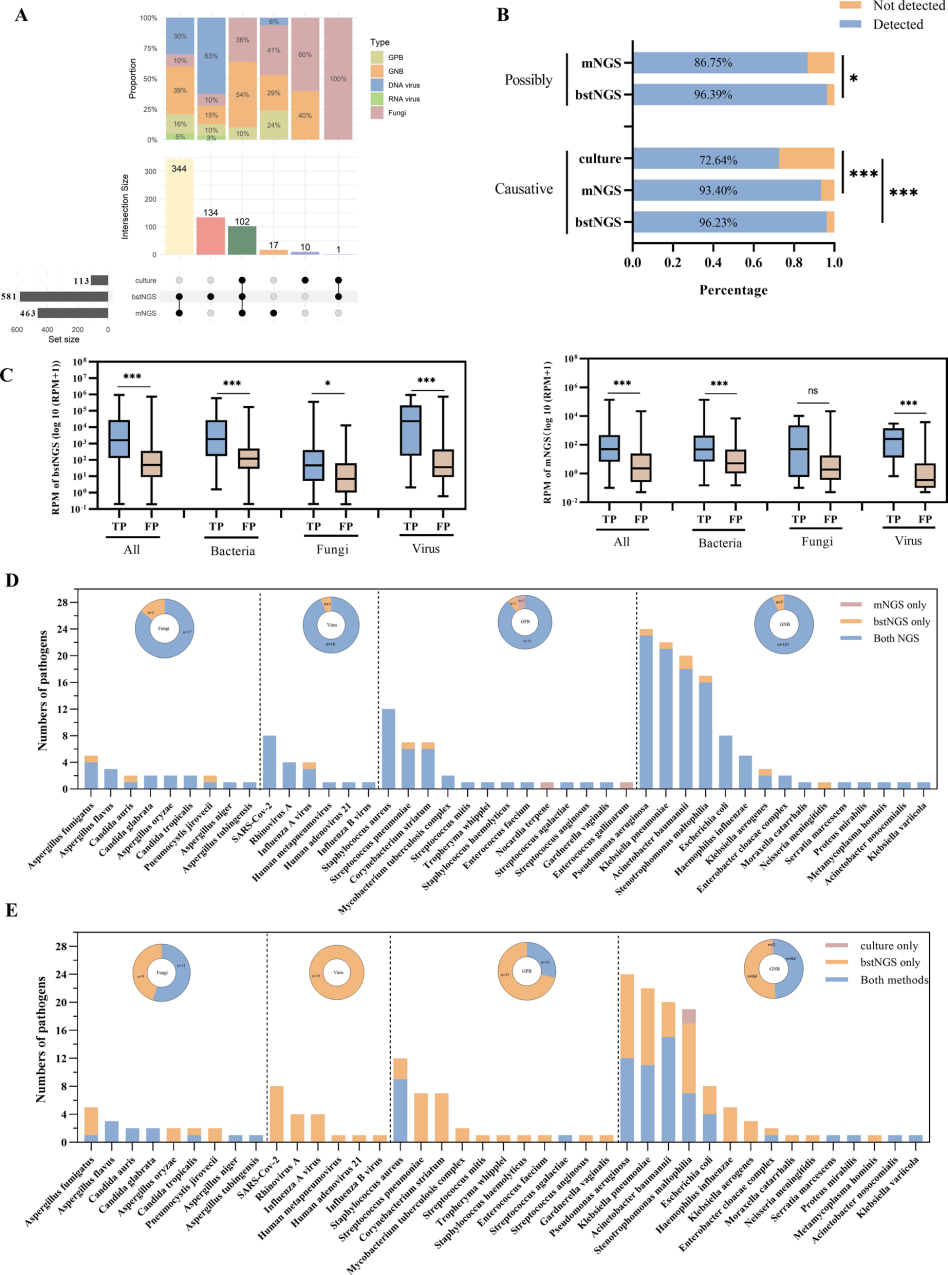
Comparison of pathogens detection between bstNGS with mNGS and culture. **A** The upset plot of microorganisms between three methods. The black solid dots below upset meaning detected in these methods. **B** The causative and possibly causative pathogen detected by three methods. **C** The comparison of normalized reads (RPM) in the causal pathogen group (TP) compared with that in the non-causal pathogen group (FP). **D** Comparison of pathogen detection between bstNGS and mNGS. The bar chart illustrates the capability of bstNGS and mNGS in detecting each pathogen. The sunburst chart summarizes the total number of detections for each taxon (bacteria, fungi, and virus) by both methods. The numbers in the chart represent the count of detections. **E** Comparison of pathogen detection between bstNGS and culture

Microorganisms were detected in 94.67% (142/150), 92.00% (138/150), and 53.33% (80/150) of the samples using bstNGS, mNGS, and culture methods, respectively. Additionally, a total of 581, 463, and 113 microorganisms were identified using bstNGS, mNGS, and culture methods, respectively. Figure 2A shows the consistency of microorganism detection among the three methods. A total of 102 microorganisms were commonly detected by all three methods, while 344 microorganisms were identified by both NGS methods. When comparing bstNGS and mNGS, all the 138 mNGS—positive samples, there is at least one microorganism that is consistent with bstNGS, and 96.33% (446/463) of the microorganisms detected by mNGS were also identified by bstNGS (Supplementary Fig. 3A). Additionally, bstNGS detected an additional 135 microorganisms compared to mNGS, mainly including bacteria and DNA viruses. Moreover, bstNGS showed a significantly higher detection rate for herpesviruses compared to mNGS (*p* < 0.05). Further analysis confirmed that the RPM values of microorganisms detected exclusively by bstNGS were significantly lower than those detected by both NGS methods simultaneously (Supplementary Fig. 3B).

Similarly, when comparing bstNGS with the culture method, in 93.75% (75/80) of the culture-positive samples were consistent with the detection results of bstNGS, and 91.15% (103/113) of the microorganisms identified by culture were also detected by bstNGS (Supplementary Fig. 4A). The RPM values of microorganisms detected exclusively through bstNGS are lower than those of microorganisms that can be cultured and detected concurrently (Supplementary Fig. 4B).

### Comparison of pathogens detection between bstNGS and other methods

Referring to the diagnostic criteria outlined in Sect. 2.4 for LRTI, a total of 189 pathogens were identified in 133 patients, including 106 causative pathogens and 83 possibly causative pathogens. The bstNGS, mNGS, and culture methods detected pathogens in 87.33% (131/150), 82.67% (124/150), and 46.00% (69/150) of the samples respectively, accounting for 31.33% (182/581), 36.93% (171/463), and 68.14% (77/113) of the detected microorganisms respectively (Supplementary Fig. 5). NGS methods demonstrated superior performance in identifying causative pathogens compared to the traditional culture method [bstNGS vs mNGS vs culture: 96.23% (102/106) vs 93.40% (99/106) vs 72.64% (77/106)]. Notably, bstNGS identified a higher number of possibly causative pathogens than mNGS [96.39% (80/83) vs 86.75% (72/83)] (Fig. 2B). Comparison of pathogens detected by NGS with non-causal microorganisms revealed significantly higher RPM values for pathogens in both bstNGS and mNGS, which somewhat eases the clinical interpretation of NGS reports (Fig. 2C).

Further comparison of pathogens detection between bstNGS and mNGS revealed that bstNGS identified slightly more pathogens than mNGS (182/189 vs 171/189, *p* = 0.023). A total of 89.42% (169/189) of pathogens were detected by both methods, with bstNGS detecting 13 additional pathogens and mNGS detecting 2 pathogens (*Nocardia terpene* and *Enterococcus gallinarum*) that were not covered by bstNGS (Fig. 2D). Both methods performed similarly in detecting fungi (20/23 vs 17/23, *p* = 0.26), Gram-positive bacteria (GPB) (35/37 vs 35/37, *p* = 1.00), and viruses (19/19 vs 18/19, *p* = 0.31), while bstNGS identified slightly more Gram-negative bacteria (GNB) (108/110 vs 101/110, *p* = 0.03). As expected, bstNGS detected more pathogens than culture (182/189 vs 77/189, *p* < 0.0001), especially viruses, fungi (20/23 vs 11/23, *p* = 0.005), GNB (108/110 vs 56/110, *p* < 0.0001), and GPB (35/37 vs 10/37, *p* < 0.0001). Despite bstNGS detecting a large number of pathogens, two cases of *Stenotrophomonas maltophilia* were only identified by culture, with secondary PCR testing confirming the presence of trace pathogen nucleic acids in the remaining samples (Fig. 2E).

### Evaluation of clinical diagnosis performance of bstNGS, mNGS, and culture

**Fig. 3:**
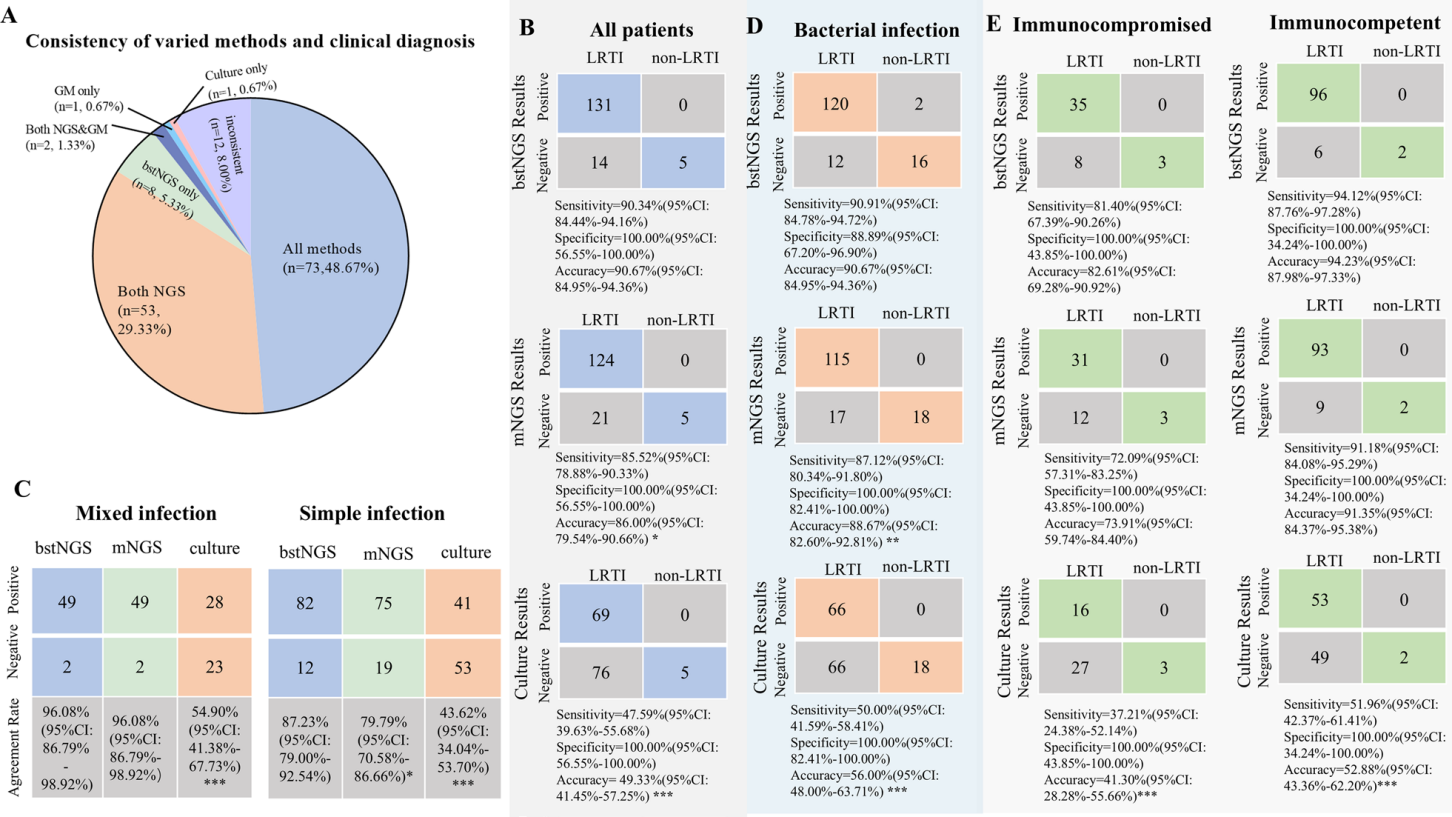
Diagnostic performance of bstNGS in the diagnosis of suspected LRTI. **A** The pie chart shows the pathogen detection capabilities of the various methods in all patients. “inconsistent” means that the detection results of the NGS method, the culture method, and the GM method were ultimately not adopted as the diagnostic results for pathogens. **B** Using a predefined composite diagnostic standard for reference, the diagnostic performance of the three methods is displayed for all patients. **C** The diagnostic agreement of the three methods for mixed infections and simple infections. **D** Using clinical diagnosis for reference, the diagnostic performance of the three methods is displayed for bacterial infections. **E** The diagnostic performance of the three methods is displayed for immunocompromised and immunocompetent patients. The differece based on the McNemar test of bstNGS and culture or mNGS was marked by *: (*p* < 0.05); **: (*p* < 0.001); ***: (*p* < 0.0001). Two times comparisons were performed, and the p-values were adjusted using the Bonferroni method to control for the type I error rate

Based on the diagnostic criteria of Sect. 2.4, 145 patients were diagnosed with LRTI, and 5 patients were diagnosed with non-infectious diseases. We first assessed the consistency of detection results among bstNGS, mNGS, and culture methods in patients suspected of LRTI to determine if they provided similar diagnostic outcomes. Overall, among the 73 samples, all three methods identified pathogens that were recognized in the clinical diagnosis or determined that there was no infection. In 55 samples, the two NGS methods detected pathogens that were incorporated into the clinical diagnosis. However, in 12 samples, the various methods did not yield results acceptable for clinical diagnosis, and ultimately, the final diagnosis for these individuals was made based on imaging findings and clinical manifestations (Fig. 3A). With clinical diagnosis as the reference (Fig. 3B), the sensitivities of bstNGS, mNGS, and culture were 90.34%, 85.52%, and 47.59%, respectively. Diagnostic accuracy was highest for bstNGS at 90.67%, slightly surpassing mNGS at 86.00% (adj. *p* = 0.046), and significantly higher than the culture method at 49.33% (adj. *p* < 0.0001) (Fig. 3B). The culture method yielded positive results consistent with clinical diagnoses in 69 samples (sensitivity: 47.59%), and bstNGS was consistent with clinical diagnoses in 97.10% (67/69) of them. The mNGS method had positive results consistent with clinical diagnoses in 124 samples (sensitivity: 85.52%), and bstNGS was 100% consistent (124/124) with clinical diagnoses (Fig. 3B; Supplementary Fig. 6A). Furthermore, the diagnostic performance of the three methods for mixed infections and simple infections was analyzed. Both bstNGS and mNGS were able to detect 96.08% (49/51) of mixed infections, which was significantly higher than the 54.90% (28/51) detected by culture (adj. *p* < 0.0001). The same results were replicated in the cases of simple infections (Fig. 3C). A detailed analysis revealed that the NGS methods demonstrated significantly better diagnostic performance than culture in cases of mixed infections involving multiple bacteria or a combination of bacteria and viruses (adj. *p* < 0.001) (Supplementary Fig. 6B). However, there was little difference in the diagnostic performance between the NGS methods and culture for mixed infections involving fungi and bacteria (Supplementary Fig. 6 C). When comparing diagnostic performance for different pathogen types, bstNGS showed better diagnostic accuracy for bacterial infections (bstNGS vs mNGS: 90.67% vs 88.67%, adj. *p* = 0.016; bstNGS vs culture: 90.67% vs 56.00%, adj. *p* < 0.0001), but its performance in fungal and viral infections was comparable to the other methods (Fig. 3D; Supplementary Fig. 6C). Both in immunocompromised and immunocompetent patients, bstNGS and mNGS demonstrated comparable diagnostic sensitivity and accuracy, which are higher than the diagnostic performance of culture—based methods (Fig. 3E).

### Comparison of bstNGS and GM for *Aspergillus* spp. detection

The GM test is commonly used for detecting *Aspergillus* spp. in BALF. We compared its detection ability with bstNGS. The GM test was positive in 16 samples, while bstNGS detected *Aspergillus* spp. in 10 (Supplementary Fig. 6D). Both methods agreed in 6 samples, resulting in a 90.67% (136/150) consistency rate. After considering symptoms, 11 patients were diagnosed with probable *Aspergillus* infection. Sensitivity for bstNGS and GM was 72.73% and 81.82%, respectively, while specificity was 98.56% for bstNGS and 94.96% for GM.

### Determinants of pathogen detection efficiency by bstNGS

**Fig. 4:**
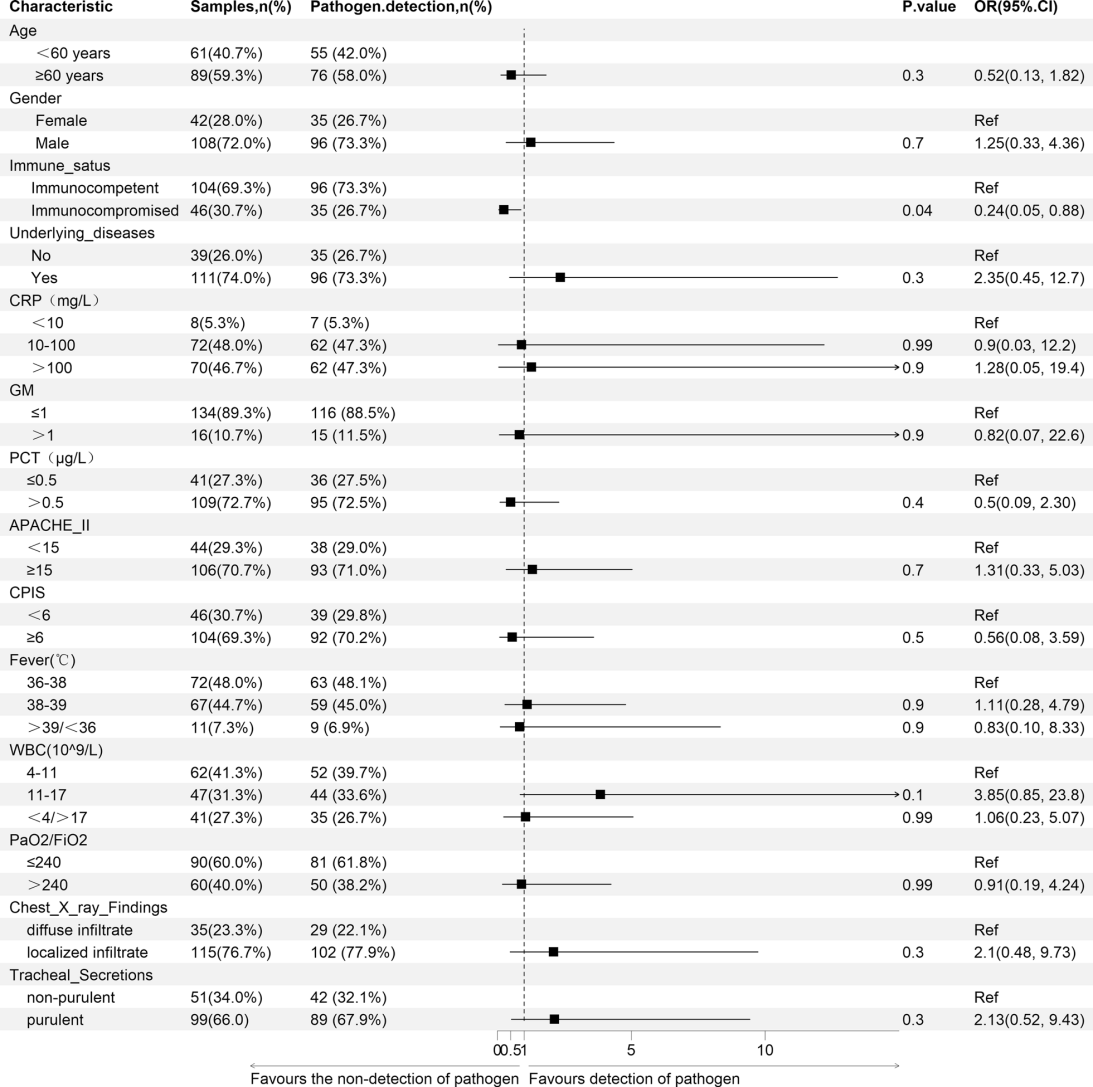
Determinants of causative or possibly causative pathogen detection by bstNGS. Association among categorical variables and causative pathogen detection by bstNGS was assessed by multivariate logistic-regression analysis. Only p values less than 0.05 were considered significant and are indicated by red dots. CPIS: Clinical pulmonary infection score. Based on the CPIS scoring results, Temperature, White Blood Cell (WBC) count, Oxygenation, Chest X-ray findings, and Tracheal secretion are assigned different point values

To assess factors influencing the detection of causative or possibly causative pathogens by bstNGS, we calculated odds ratios (ORs) using multivariate logistic regression (Fig. 4; Supplementary Fig. 1). The results showed that in the immunocompromised patients, the proportion of identifying the pathogens by bstNGS was lower, and no differences were found in other subgroups. Although there was a trend suggesting that the subgroup with elevated WBC count might have a higher detection potential, there was no significant difference. Similar results were also found in the analysis of mNGS (Supplementary Table 2). The difference was that not only the patient’s immune status but also the elevated WBC count was associated with the detection efficiency of mNGS.

### Association between outcome and pathogen detection by bstNGS

**Fig. 5:**
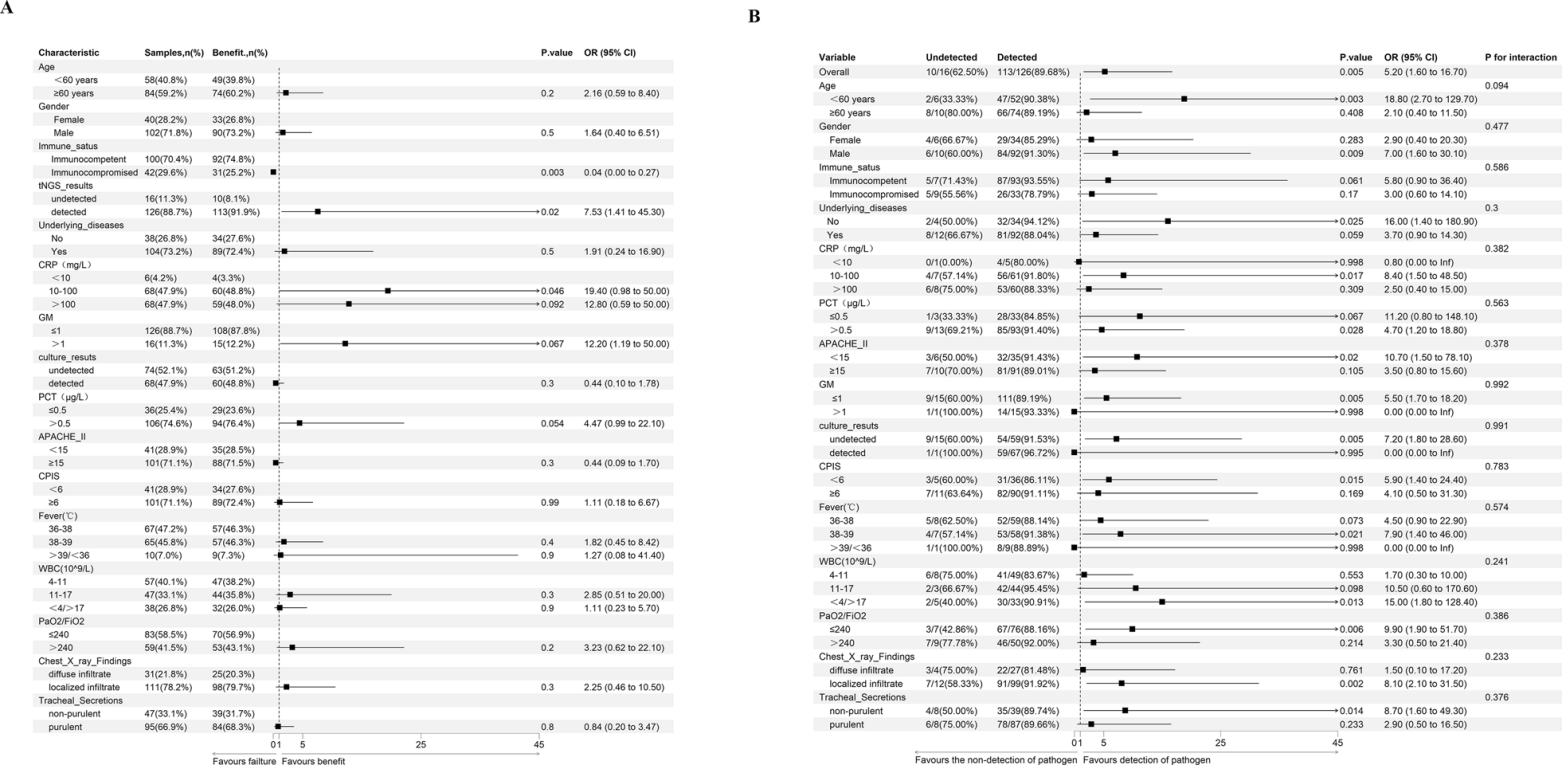
Association between Outcome and pathogen detection by bstNGS. **A** multivariate logistic-regression analysis for outcome and pathogen detection by bstNGS. **B** Subgroup analysis of the association between Outcome and pathogen detection by bstNGS. Only *p* values less than 0.05 were considered significant and are indicated by red dots. CPIS: Clinical pulmonary infection score. Based on the CPIS scoring results, Temperature, White Blood Cell (WBC) count, Oxygenation, Chest X-ray findings, and Tracheal secretion are assigned different point values

Outcomes of 7-day antibiotic treatment were assessable in 142 patients. Among those, 86.62% (123/142) benefited from antibiotic treatment. Of those who benefited, 91.87% (113/123) had causative or possibly causative pathogens detected by bstNGS. In contrast, among patients with treatment failure, pathogens were identified in 68.42% (13/19) cases. We assessed the relationship between pathogen detection by bstNGS and patient outcomes using multivariate logistic regression (Fig. 5A; Supplementary Fig. 1). Compared to the group without pathogen detection, the group with pathogen detection by bstNGS had a significantly higher rate of benefit (89.68% vs 62.50%; OR 7.53, 95% CI 1.41–45.30; *p* = 0.02). The same results were also observed in the mNGS detection group (OR 7.41, 95% CI 1.30–50.61; *p* = 0.029; Supplementary Table 3). Meanwhile, we observed that the benefit rate of immunocompromised patients was significantly lower (73.81% vs 92.00%; OR 0.04, 95% CI 0.0001–0.270; *p* = 0.003). In contrast, the benefit rate was higher in the groups with elevated C-reactive protein (CRP) and procalcitonin (PCT) levels. Further exploratory subgroup analysis indicated that the correlation between the detection of pathogens by bstNGS and a better prognosis was consistent across all subgroups, including those with different immune statuses, as well as subgroups with different CRP and PCT levels (Fig. 5B).

## Discussion

Targeted NGS (tNGS) has garnered significant attention as a promising auxiliary diagnostic method due to its performance and potential value in clinical workflows. Although several retrospective studies have assessed the diagnostic performance of tNGS in respiratory infections [13–15, 25], prospective studies exploring the clinical applicability of this method remain scarce. In this study, we conducted a prospective evaluation of the diagnosis of ICU patients using bstNGS. The results showed that bstNGS demonstrated diagnostic accuracy comparable to that of mNGS, and was significantly superior to culture. Additionally, our results address several clinically relevant questions.

Targeted pathogen enrichment is an emerging approach [12, 28, 29], though its clinical performance, especially in respiratory sample detection, remains to be fully established. Our head-to-head comparison of bstNGS with other diagnostic methods revealed excellent consistency with mNGS. The results of this study indicated that bstNGS is capable of detecting most of the microorganisms discovered by both mNGS (96.33%, 446/463) and culture (91.15%, 103/113) (Supplementary Figs. 3A & 4A), while also identifying a substantial number of additional microorganisms. Similar to previous studies on severe pneumonia, these additional detections were mainly due to bstNGS’s ability to enrich low-abundance microbes [13, 15, 17, 25]. It is well-established that tNGS, through probe hybridization enrichment, significantly enhances the detection of fungi, viruses, and bacteria [29, 30]. In our study, bstNGS demonstrated a notable enrichment effect across various microorganisms, particularly viruses, when compared to mNGS. These findings align with recent retrospective studies [15, 31]. Moreover, when compared to culture methods, bstNGS detected common pathogens like *Acinetobacter baumannii*, *Klebsiella pneumoniae*, and *Pseudomonas aeruginosa* more frequently. The microorganisms detected by bstNGS likely benefited from its lower limit of detection.

Another important question is whether targeted enrichment can simplify the clinical interpretation of NGS results. While tNGS has a narrower coverage range than mNGS, the number of microorganisms detected in a single test is not reduced [13–15, 17]. This is likely because tNGS covers many common respiratory commensals [14, 15, 17, 25]. Our results show that NGS methods outperform culture methods in determining causative pathogens (bstNGS vs. mNGS vs. culture: 96.23% vs. 93.40% vs. 72.64%) (Fig. 2B). Furthermore, bstNGS significantly improved the detection of possibly causative pathogens compared to mNGS (96.39% vs. 86.75%, p < 0.05) (Fig. 2B). In our cohort of 150 samples, bstNGS identified the highest number of pathogens, providing more options for clinical diagnosis. Both bstNGS and mNGS showed significantly higher RPM values for pathogens compared to other microorganisms, a trend also reported in previous studies [6, 15, 25, 29, 30]. This suggests that NGS bioinformatics parameters are, to some extent, linked to pathogen risk, which could alleviate the challenges in interpreting NGS reports.

Evaluating the performance of emerging diagnostic methods like tNGS is both essential and challenging. Several studies have retrospectively assessed tNGS’s clinical diagnostic value in comparison to mNGS [13–15]. Gaston et al. found that mNGS and tNGS exhibited similar sensitivity and specificity for clinical diagnosis [13]. Other studies confirmed comparable performance between tNGS and mNGS in severe pneumonia patients [14, 15]. tNGS demonstrated superior performance in viral detection and outperformed mNGS in diagnosing viral co-infections, likely due to its ability to detect both DNA and RNA pathogens in a single test. However, in our study, we observed no significant differences between bstNGS and mNGS in diagnosing viral co-infections, likely because our mNGS utilized RNA-based testing for all patients [14, 15]. Furthermore, bstNGS exhibited higher diagnostic accuracy than mNGS, particularly in detecting Gram-negative bacteria (GNB), likely due to its lower detection limit for these pathogens. However, for *Aspergillus* spp. detection, the GM test remains valuable, highlighting that while NGS methods are powerful, they do not always surpass traditional tests in detecting specific fungi [16, 17]. This is probably why, although bstNGS has a stronger diagnostic ability for mixed infections than culture, it is comparable to culture in the mixed infection pattern of fungi and bacteria. In addition, we observed that the turnaround time of bstNGS is approximately 16–18 h, which is comparable to the 22–26 h of mNGS. However, in terms of cost, bstNGS only costs half as much as mNGS. This makes bstNGS more likely to have the potential to be used in combination with traditional microbiological tests.

Prospective studies are needed to fully understand the clinical implications of tNGS and its impact on patient outcomes. In our cohort, we analyzed the factors influencing the detection of pathogens by bstNGS. We found that bstNGS was affected in immunocompromised patients, and no factors influencing its performance were identified in other subgroups of the included patient statuses [17, 24]. This indicates that bstNGS can be regarded as a first-line or second-line diagnostic tool for infections in ICU patients. However, for immunocompromised patients, extra attention should be paid to whether to choose this method. This study demonstrated that bstNGS exhibited higher diagnostic accuracy than culture in both immunocompromised and immunocompetent patients, consistent with prior research [17, 32]. Notably, bstNGS showed a higher pathogen detection rate among immunocompetent ICU patients, diverging from most existing studies. However, similar findings were reported in several investigations, such as Liu’s meta-analysis (92% vs 90% diagnostic accuracy in immunocompetent vs immunocompromised individuals) and Sun’s study (91% vs 83%) [32, 33]. Currently, no significant differences in pathogen detection rates between the two groups have been reported. Several factors may account for our results. Firstly, the proportion of patients infected with specific pathogens plays a role. Previous studies indicated that mNGS was superior in diagnosing *Pneumocystis jirovecii* but less effective for *Aspergillus* compared to traditional methods [16, 17]. Given the higher prevalence of Aspergillus infections in immunocompromised patients, its proportion in small-scale evaluations can influence mNGS performance, as exemplified by an immunocompromised patient with *Aspergillus* infection diagnosed solely by the GM test in our study. Secondly, as a prospective study, it included 12 patients (6 in the immunocompromised group) with infections of undetermined pathogen, which may have affected NGS detection rates across subgroups. Finally, patient population composition matters. Studies show that mNGS performance does not differ between immunocompetent and immunocompromised HIV-infected patients [24], while its diagnostic accuracy is lower in solid organ transplant recipients than other immunocompromised patients [34]. Additionally, mNGS performs better in immunocompromised patients from infectious disease departments but similarly in ICU-admitted immunocompetent and immunocompromised patients [35]. Our immunocompromised cohort, mainly composed of solid tumor patients under treatment, may thus impact pathogen detection outcomes. This result requires further exploration in a larger population or with a focus on this specific population. Additionally, we explored the relationship between bstNGS pathogen detection and antibiotic treatment outcomes, finding that the proportion of patients benefiting from bstNGS results was significantly higher, supporting similar conclusions from previous clinical studies [15, 17, 25], and further subgroup analyses reinforced this finding. These results offer theoretical support for future studies on the clinical utility of bstNGS in guiding treatment decisions. We speculate that this benefit may stem from the ability of NGS to enhance etiological diagnosis and inform anti-infective therapy. However, as our study is observational, larger, well-designed randomized controlled trials (RCTs) are needed to more definitively assess the impact of bstNGS on clinical outcomes.

Our study has several limitations. Firstly, there are certain limitations in the design of this study. It is a single-center prospective study with a relatively small sample size and a limited range of patients. This makes it impossible to effectively avoid the potential influence of confounding factors such as population characteristics. It is necessary to validate the study results through multi-center and larger population cohorts. Secondly, since it is an observational study, the relationship between bstNGS results and the prognosis of patients under antibiotic treatment needs to be confirmed through further interventional studies. Thirdly, the diagnostic reference criteria adopted in this study incorporated the results of NGS testing, which may potentially lead to biases in the reference diagnosis. To mitigate these biases, we adopted the diagnostic criteria from previous NGS evaluation studies. This set of criteria encompasses patients' clinical manifestations, NGS results, other laboratory test results, and imaging findings. Moreover, we also implemented a dual independent evaluation approach. Lastly, we did not include quantitative PCR methods to assess DNA virus infections, which limited our ability to evaluate the detection of human herpesvirus infections by bstNGS.

## Conclusion

In summary, our prospective study confirms that the bstNGS method shows high diagnostic performance than mNGS and culture methods, with enhanced potential for bacterial detection. In clinical practice, apart from the need to pay attention to immunocompromised patients, the efficiency of bstNGS in detecting pathogens is similar among most patients. Moreover, for patients in whom pathogens are detected by bstNGS, better clinical outcomes may be achieved through auxiliary-guided treatment.

## Supplementary Information

Supplementary Material 1

Supplementary Material 2

Supplementary Material 3

Supplementary Material 4

## Data Availability

The data that support the findings of this study are available from the corresponding author on request.

## Abbreviations

tNGS: Targeted next-generation sequencing
bstNGS: Broad-spectrum targeted next-generation sequencing
LRTI: Lower respiratory tract infections
mNGS: Metagenomic next-generation sequencing
BALF: Bronchoalveolar lavage fluid
PPV: Positive predictive value
NPV: Negative predictive value
CMT: Conventional microbiological test
ICU: Intensive care units
RPM: Read per million
GPB: Gram-positive bacteria
GNB: Gram-negative bacteria
GM: Galactomannan
