# Supplementary material for: Performance of broad-spectrum targeted next-generation sequencing in lower respiratory tract infections in ICU patients: a prospective observational study
Source: Crit Care. 2025 Jun 4;29:226. doi: 10.1186/s13054-025-05470-z (PMC12139122; doi:10.1186/s13054-025-05470-z)
Supplement: Supplementary file 1 — Supplementary Material 1 [file 13054_2025_5470_MOESM1_ESM.docx]

**Supplementary Figure 1.**


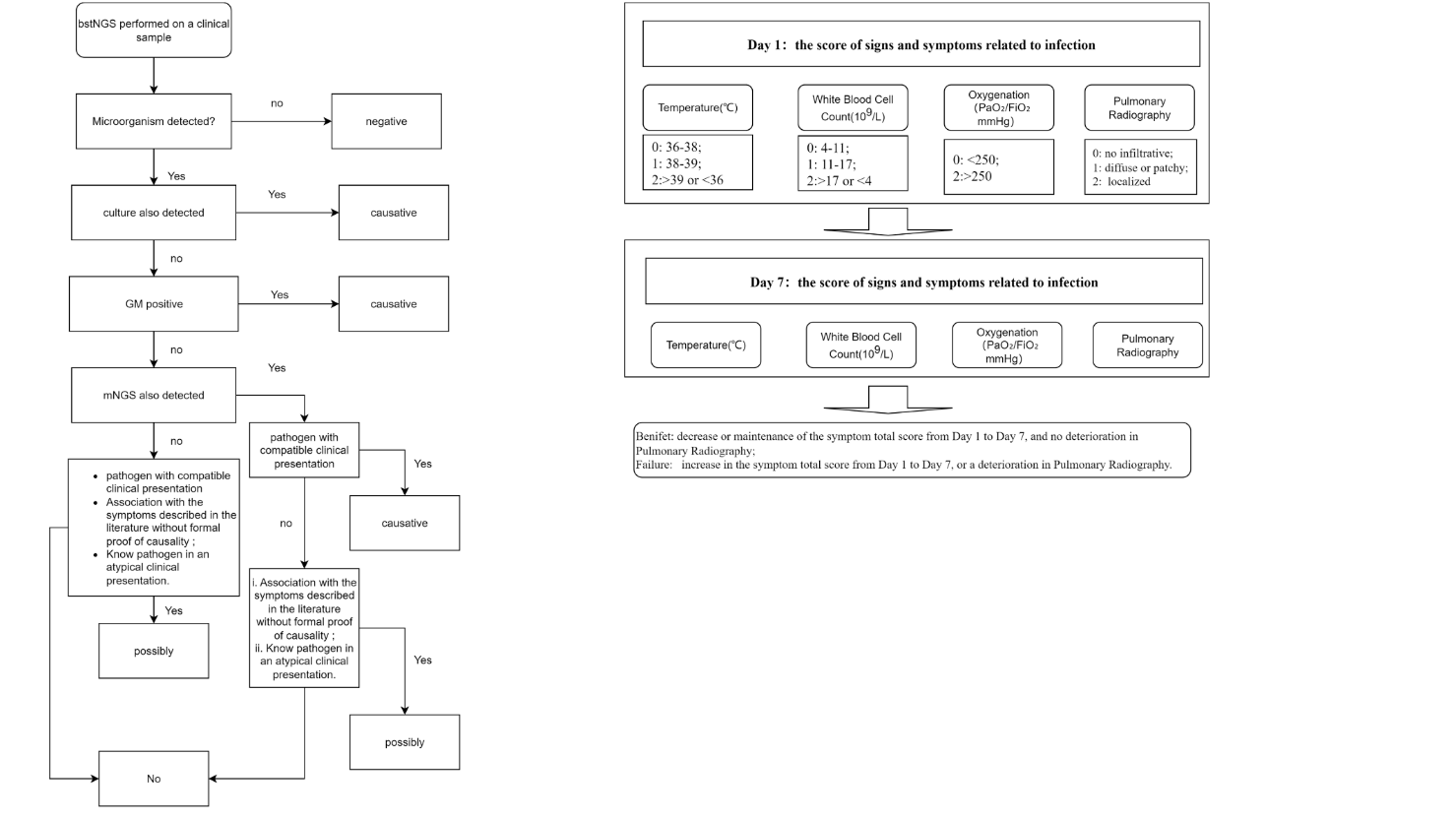


**Supplementary Figure 2.**


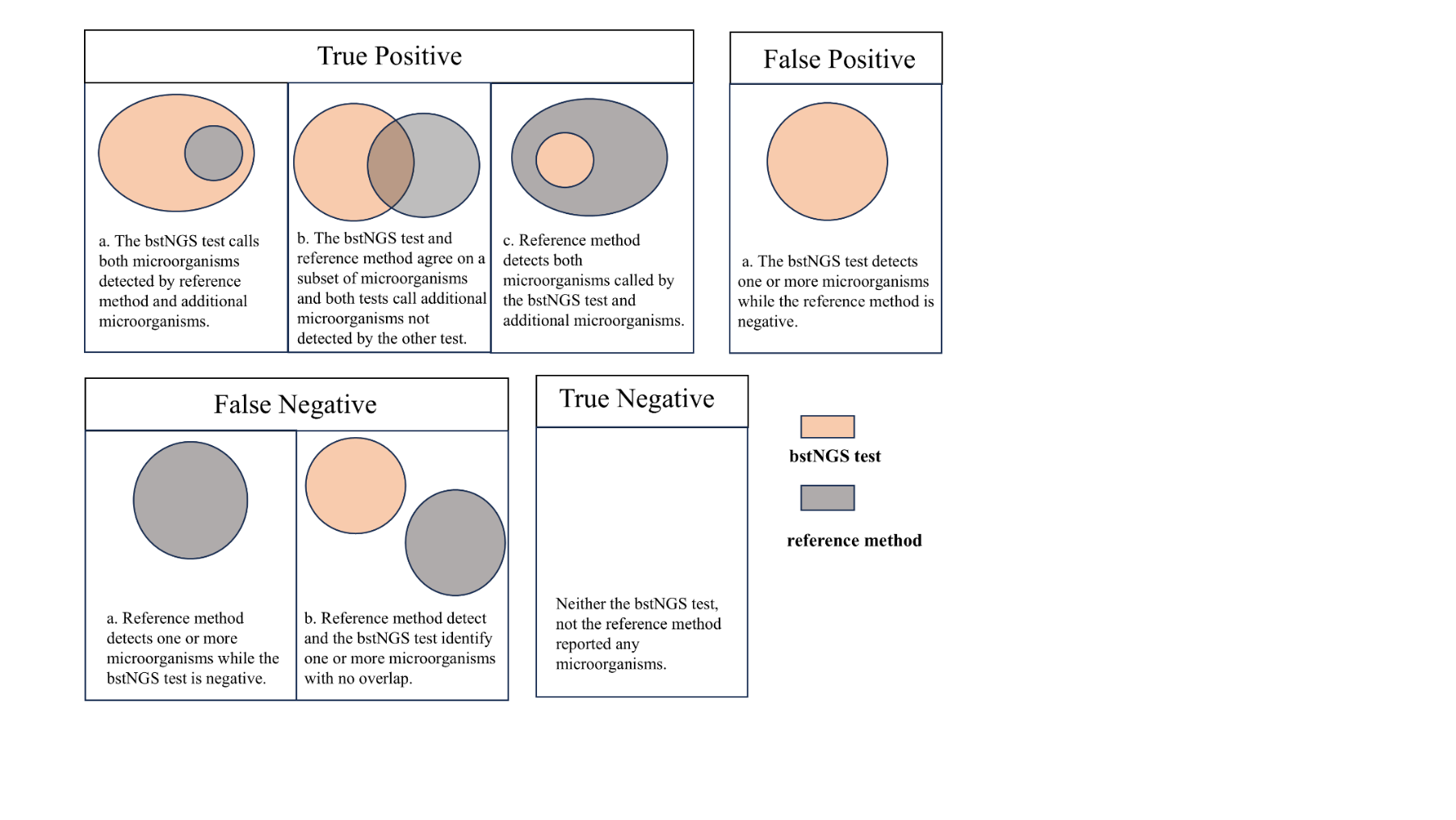


**Supplementary Figure 3.**


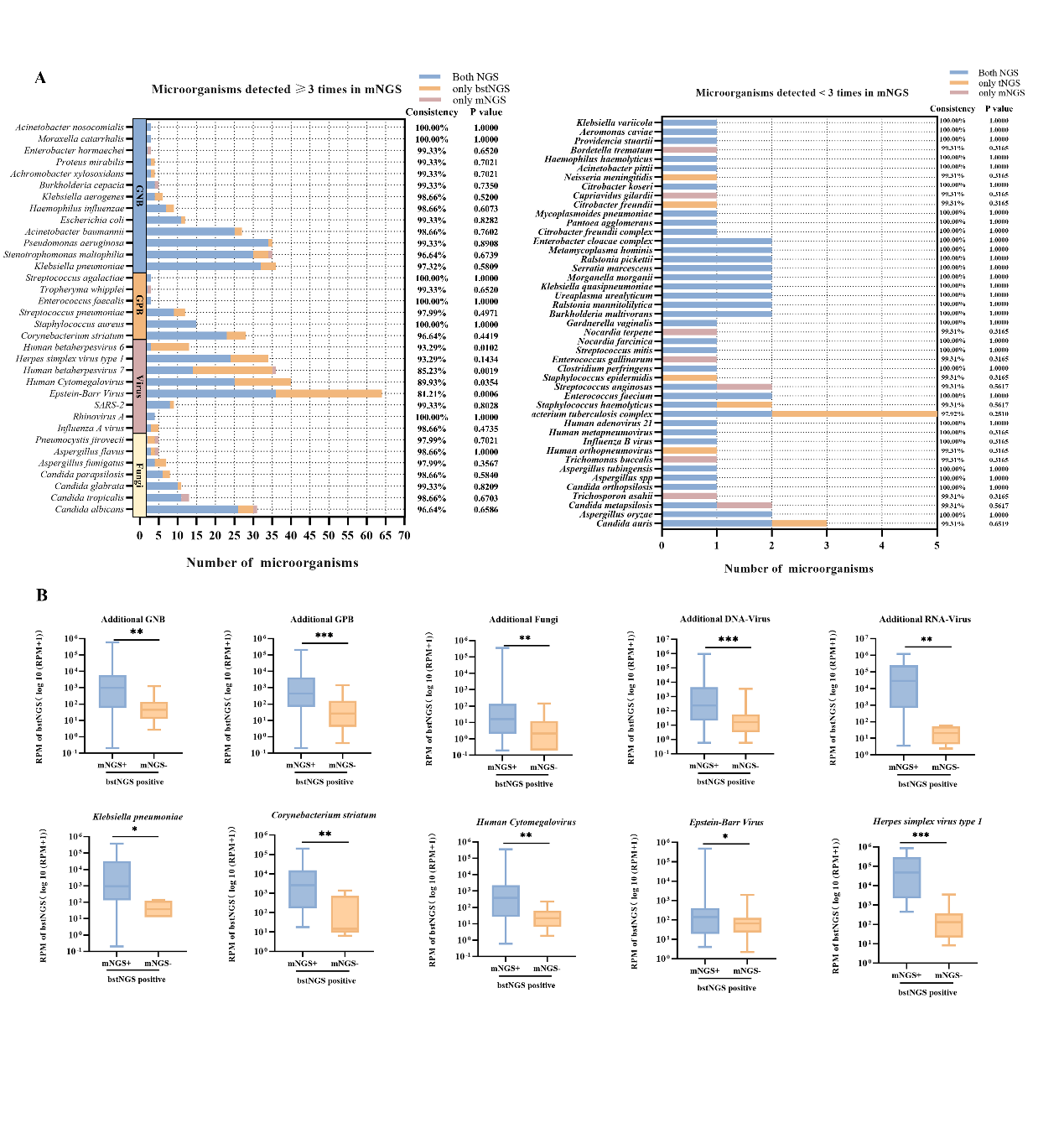


**Supplementary Figure 4.**


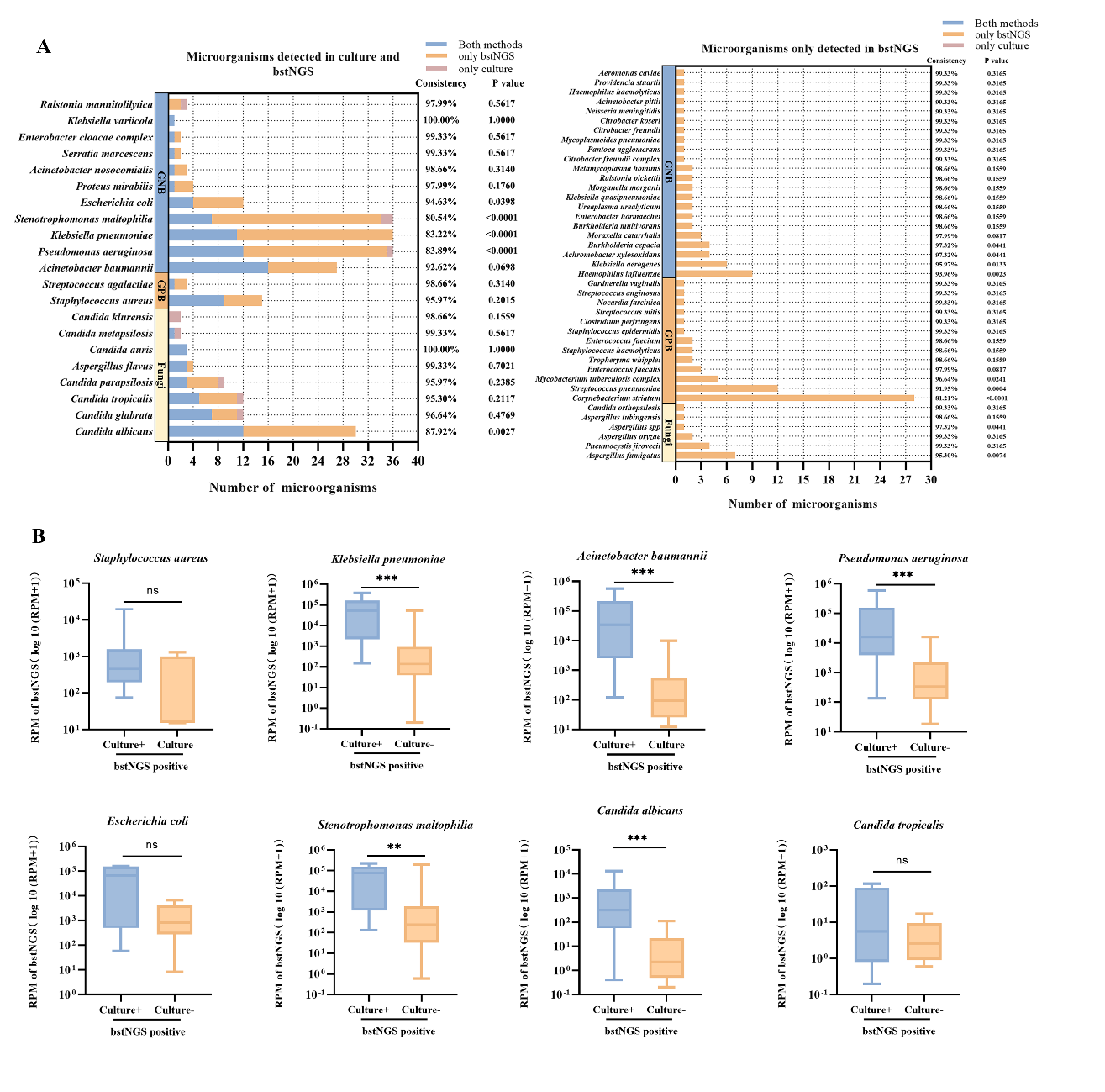


**Supplementary Figure 5.**


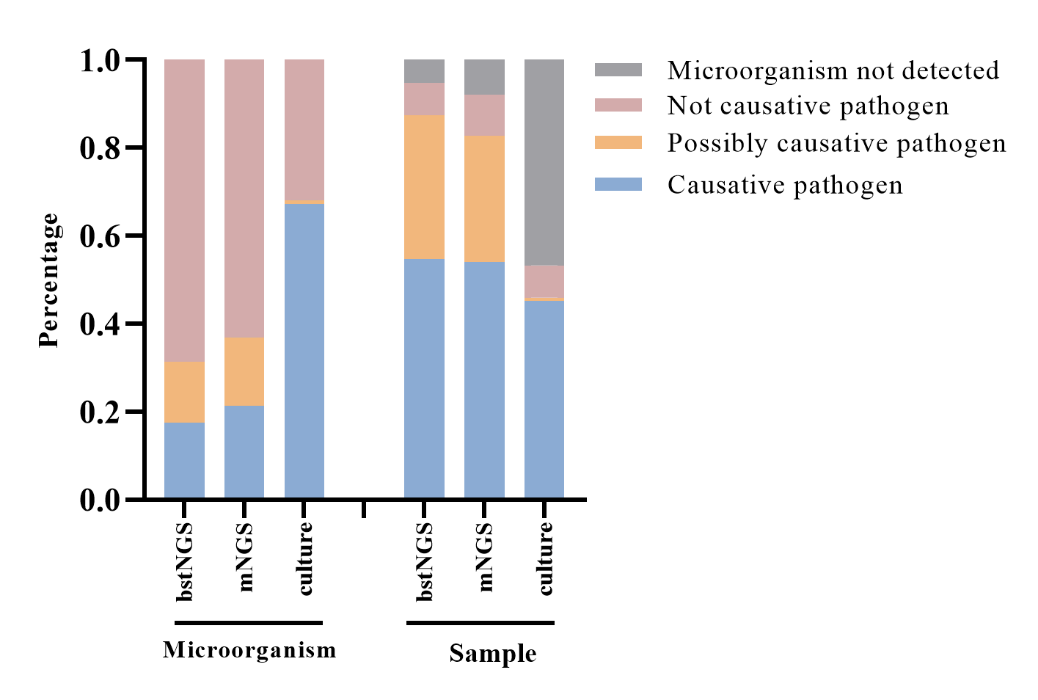


**Supplementary Figure 6.**


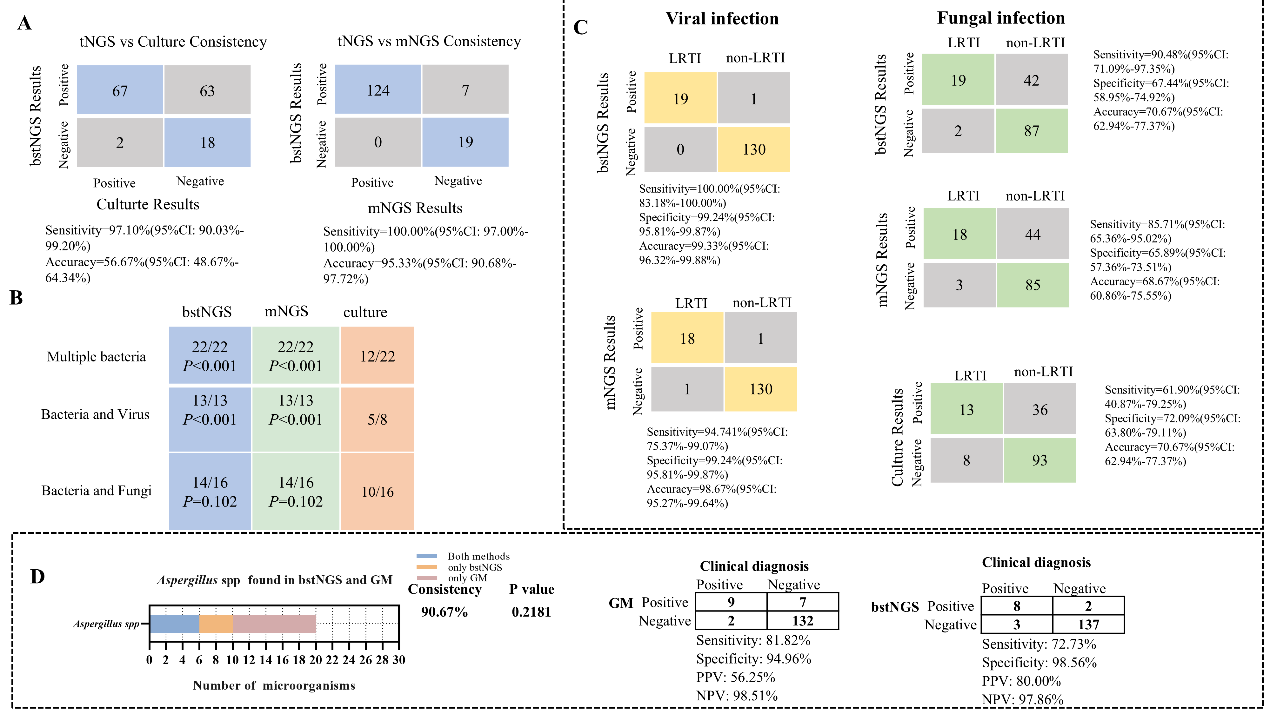


**Supplementary Figure 1**. Flowchart of pathogenic microorganisms identifying and outcomes of antibiotic treatment. The day of admission to the ICU is considered as Day 1.

**Supplementary Figure 2**. Scoring structure for bstNGS test comparisons to reference method (culture/mNGS/clinical diagnosis).

**Supplementary Figure 3**. (A). The consistency of bstNGS with mNGS in microorganisms detected in mNGS. Consistency ratios were identified by numbers. GPB: Gram-positive bacteria. GNB: Gram-negative bacteria. Consistency was calculated as follows: consistency = (Total - only bstNGS - only mNGS) / Total. (B). Further analysis to additional detections by RPM of bstNGS. The results were grouped based on whether the mNGS was positive or negative (labeled as mNGS+ or mNGS-). *: *p* < 0.05, **: *p* < 0.01, ***: *p* < 0.001.

**Supplementary Figure 4**. (A)**.** Evaluation of the concordance of bstNGS to culture in microorganisms identified in culture. Concordance ratios were identified by numbers. GNB: Gram-negative bacteria. (B). Comparison analysis of RPM in bstNGS for microorganisms that consistency ≤ 95%. The results were grouped based on whether the culture results were positive or negative (labeled as culture+ or culture-).

**Supplementary Figure 5**. The detection of causative and possibly causative pathogens in three methods.

**Supplementary Figure 6**. The diagnostic performance of the different methods. (A). Taking the samples with pathogens detected by culture and mNGS as references respectively, the consistency of bstNGS with them (sensitivity and accuracy). (B). The detection consistency of the three methods with clinical diagnosis in different mixed - infection patterns. (C) The diagnostic performance of the different methods in viral and fungal infections. (D). Comparison analysis of detection of *aspergillus* between GM and bstNGS.
