# Supplementary material for: Performance of broad-spectrum targeted next-generation sequencing in lower respiratory tract infections in ICU patients: a prospective observational study
Source: Crit Care. 2025 Jun 4;29:226. doi: 10.1186/s13054-025-05470-z (PMC12139122; doi:10.1186/s13054-025-05470-z)
Supplement: Supplementary file 2 — Supplementary Material 2 [file 13054_2025_5470_MOESM2_ESM.docx]

Supplementary methods

1. broad-spectrum tNGS assay workflow

1.1 Sample processing and nucleic acid extraction

BALF was divided into aliquots of 400 μL for nucleic acid extraction. Samples, and lysis buffer(Vazyme, Nanjing, China), protease K mixture, binding buffer (Vazyme, Nanjing, China), and 1.2 g glass bead were added to the grinding tube and agitated vigorously at 4500 rpm for a total of 30s by FastPrep-24™ 5G Instrument (MP Biomedical, CA, USA). Samples rested at room temperature for approximately 20 min before nucleic acid extraction. Extraction was performed using the VAMNE Magnetic Pathogen DNA/RNA kit (Vazyme, Nanjing, China). Nucleic acids were quantified using a Qubit 3.0 fluorometer with double-stranded DNA (dsDNA) and RNA high-sensitivity (HS) reagents. Then, the RNA was reverse-transcribed into cDNA using Hieff NGS® ds-cDNA Synthesis Kit (Yeasen, Shanghai, China). A549 human cells (GenePlus, Suzhou, China) were used as negative controls (NTC) to detect contamination, and A549 human cells spiked with Staphylococcus aureus (BeNa Culture Collection, Beijing, China) were used as positive controls (PTC).

1.2 Library preparation and enrichment

Load 50 ng of the extracted nucleic acid based on its concentration. cDNA synthesis and library preparation were performed with the HieffNGS®C37P4 One PotcDNA&gDNA Library Prep Kit (Yeasen, Shanghai, China) according to the protocol. An aliquot was taken through library enrichment with NadPrep® NanoBlockers (Nanodigmbio, Nanjing, China) reagents to generate the product for targeted sequencing, with the remaining saved after the post-indexing cleanup step as the product for metagenomic sequencing. Target enrichment was performed by incubating Geneplus probes (covered 1872 species) with samples for approximately 4 h. The probe and bound products were captured, removed, and eluted and underwent an 18-cycle PCR for library preparation. Products for metagenomic and targeted sequencing were quantified with a Qubit 3.0 instrument using dsDNA HS reagents. Products were stored at −20°C until sequencing.

1.3 Sequencing and Bioinformatic analysis

Sequencing was performed on Gene+Seq-100 (GenePlus-Suzhou, China) with a 100-bp single-end read sequencing to goal depths of 5 million reads for the targeted workflow. After sequencing, ensure that the final sequencing output is no less than 5 million reads, with Q20 and Q30 values not lower than 95% and 88%, respectively. Clean reads were obtained by removing sequencing adapters, low-quality reads, or reads below 35 bp using fastp (version 0.23.1). The remaining reads were aligned to the human reference (hg38) using Burrow-Wheeler Aligner (version 0.7.12-r1039), and human reads were filtered. The filtered reads were compared with the self-built pathogenic microorganism database, and the retained results were annotated. Analysis of sequencing data generated by the workflow was a Geneplus' self-built automated Data Analysis Solution. Microbial reads within the target range were normalized to reads per million (RPM), and only microorganisms above a predefined threshold were initial reported in this study. The threshold was set at RPM ≥6 for common pathogens (excluding mycobacteria) and ≥0.5 for fungi and mycobacteria. A manual review is conducted. Oral commensals were not reported regardless of their relative abundance, unless otherwise proven or deemed significant by the attending physician. Microorganisms with abnormal genomic coverage will be filtered out.

Upon arrival at the laboratory within 1 hour, the median turnaround time for mNGS was 20 hours (interquartile range: 19-25 hours; maximum: 28 hours), whereas for bstNGS, it was 18 hours (interquartile range: 16-21 hours; maximum: 24 hours), indicating a comparable duration for both sequencing processes.
