# Supplementary material for: Performance of broad-spectrum targeted next-generation sequencing in lower respiratory tract infections in ICU patients: a prospective observational study
Source: Crit Care. 2025 Jun 4;29:226. doi: 10.1186/s13054-025-05470-z (PMC12139122; doi:10.1186/s13054-025-05470-z)
Supplement: Supplementary file 4 — Supplementary Material 4 [file 13054_2025_5470_MOESM4_ESM.docx]

**Supplementary Table 2.** Determinants of causative or possibly causative pathogen detection by mNGS

| **Characteristic** | **p.value** | **OR (95% CI)** |
| --- | --- | --- |
| Age |  |  |
| ＜60 years |  |  |
| ≥60 years | 0.3 | 0.48(0.13, 1.62) |
| Gender |  |  |
| Female |  |  |
| Male | 0.5 | 1.55(0.46, 5.14) |
| Immune_satus |  |  |
| Immunocompetent |  |  |
| Immunocompromised | 0.024 | 0.24(0.07, 0.80) |
| Underlying_diseases |  |  |
| No |  |  |
| Yes | >0.9 | 0.97(0.19, 4.37) |
| CRP（mg/L） |  |  |
| ＜10 |  |  |
| 10-100 | 0.4 | 3.03(0.23, 34.0) |
| ＞100 | 0.5 | 2.44(0.16, 30.1) |
| GM |  |  |
| ≤1 |  |  |
| ＞1 | 0.4 | 3.07(0.30, 79.2) |
| culture_resuts |  |  |
| undetected |  |  |
| detected | 0.002 | 31.1(5.34, 610) |
| PCT（μg/L） |  |  |
| ≤0.5 |  |  |
| ＞0.5 | 0.6 | 0.65(0.14, 2.75) |
| APACHE_II |  |  |
| ＜15 |  |  |
| ≥15 | 0.7 | 1.31(0.38, 4.48) |
| CPIS |  |  |
| ＜6 |  |  |
| ≥6 | 0.8 | 0.87(0.25, 3.15) |
| Fever(℃) | 0.8 | 1.26(0.16, 13.0) |
| 36-38 |  |  |
| 38-39 |  |  |
| ＞39/＜36 | 0.5 | 1.88(0.35, 11.0) |
| WBC (10^9/L) |  |  |
| 4-11 |  |  |
| 11-17 | 0.044 | 4.41(1.14, 21.5) |
| ＜4/＞17 | 0.6 | 1.53(0.35, 7.14) |
| PaO2/FiO2 |  |  |
| ≤240 |  |  |
| ＞240 | 0.3 | 0.49(0.11, 1.92) |
| Chest_X_ray_Findings |  |  |
| diffuse infiltrate |  |  |
| localized infiltrate | 0.8 | 0.87(0.22, 3.39) |
| Tracheal_Secretions |  |  |
| non-purulent |  |  |
| purulent | 0.3 | 2.01(0.55, 7.88) |

**Supplementary Table 3.** multivariate logistic-regression analysis for outcome and pathogen detection by mNGS.

| **Characteristic** | **P.value** | **OR (95%CI)** |
| --- | --- | --- |
| Age |  |  |
| ＜60 years |  |  |
| ≥60 years | 0.2 | 2.59(0.70, 10.6) |
| Gender |  |  |
| Female |  |  |
| Male | 0.4 | 1.87(0.47, 7.39) |
| Immune_satus |  |  |
| Immunocompetent |  |  |
| Immunocompromised | 0.003 | 0.04(0.00, 0.26) |
| mNGS_results |  |  |
| undetected |  |  |
| detected | 0.029 | 7.41(1.30, 50.6) |
| Underlying_diseases |  |  |
| No |  |  |
| Yes | 0.4 | 2.21(0.28, 19.9) |
| CRP（mg/L） |  |  |
| ＜10 |  |  |
| 10-100 | 0.034 | 21.1(1.18, 407) |
| ＞100 | 0.06 | 16(0.82, 331) |
| GM |  |  |
| ≤1 |  |  |
| ＞1 | 0.083 | 10.2(1.04, 275) |
| culture_resuts |  |  |
| undetected |  |  |
| detected | 0.2 | 0.34(0.06, 1.54) |
| PCT (μg/L) |  |  |
| ≤0.5 |  |  |
| ＞0.5 | 0.083 | 3.63(0.84, 16.4) |
| APACHE_II |  |  |
| ＜15 |  |  |
| ≥15 | 0.3 | 0.46(0.10, 1.76) |
| CPIS |  |  |
| ＜6 |  |  |
| ≥6 | >0.9 | 1.12(0.18, 6.72) |
| Fever (℃) |  |  |
| 36-38 |  |  |
| 38-39 | 0.4 | 1.89(0.47, 8.78) |
| ＞39/＜36 | 0.9 | 0.8(0.05, 24.8) |
| WBC (10^9/L) |  |  |
| 4-11 |  |  |
| 11-17 | 0.5 | 1.87(0.32, 12.6) |
| ＜4/＞17 | 0.8 | 0.83(0.17, 4.02) |
| PaO2/FiO2 |  |  |
| ≤240 |  |  |
| ＞240 | 0.2 | 3.32(0.65, 22.2) |
| Chest_X_ray_Findings |  |  |
| diffuse infiltrate |  |  |
| localized infiltrate | 0.2 | 2.53(0.52, 12.1) |
| Tracheal_Secretions |  |  |
| non-purulent |  |  |
| purulent | 0.6 | 0.72(0.17, 2.92) |
